# Psychosocial risk factors for obesity among women in a family planning clinic

**DOI:** 10.1186/1471-2296-5-20

**Published:** 2004-09-20

**Authors:** James E Rohrer, Barbara M Rohland

**Affiliations:** 1 Department of Family and Community Medicine Texas Tech University Health Sciences Center, USA; 2 Associate Professor and Regional Chair of the Department of Psychiatry (Amarillo), Texas Tech University Health Sciences Center, 1400 Coulter Blvd, Amarillo TX 79106, USA

## Abstract

**Background:**

The epidemiology of obesity in primary care populations has not been thoroughly explored. This study contributes to filling this gap by investigating the relationship between obesity and different sources of personal stress, mental health, exercise, and demographic characteristics.

**Methods:**

A cross-sectional survey using a convenience sample. Five hundred women who attended family planning clinics were surveyed and 274 provided completed answers to all of the questions analyzed in this study. Exercise, self-rated mental health, stress, social support, and demographic variables were included in the survey. Multiple logistic regression analysis was performed.

**Results:**

After adjusting for mental health, exercise, and demographic characteristics of subjects, analysis of the data indicated that that being having a large family and receiving no support from parents were related to obesity in this relatively young low-income primary care sample, but self-reported stress and most types of social support were not significant.

**Conclusion:**

Obesity control programs in primary care centers directed at low-income women should target women who have large families and who are not receiving support from their parents.

## Background

Many variables may influence eating behavior and therefore may also influence obesity, including depression, anxiety, stress, social support, race, ethnicity, education and income [1-8]. While the national media and federal websites emphasize the importance of physical activity in controlling body weight, exercise alone is not effective for this purpose. In fact, some research shows no relationship between exercise and body weight in community samples [1]. Therefore, the important questions are: what other risk factors in addition to exercise may affect obesity and how can they be changed?

Studies of stress as a risk factor for obesity are limited. In fact, few epidemiological studies have been reported in primary care journals. An exception is found in a report by Sammel et al, who included a stress index in their study of weight gain among women in their late reproductive years (ages 35–47) [1]. Three hundred and thirty-six women were followed for four years. A 14-item Perceived Stress Scale was used to assess the degree to which situations were stressful to the subject. Women who gained more than ten pounds were not different in regard to this stress measure than other subjects.

Stress can be measured in different ways. The personal circumstances experienced by individuals might be expected to have a direct effect on depression, anxiety, and general health status. After all, when conflict arises within a family, the psychological consequences can be dramatic. Even though the relevance of various sources of personal stress to obesity has not previously been examined in the community health literature, their potential importance is worthy of investigation.

The purpose of the study reported here was to investigate the importance of personal stressors in determining obesity. The sample was drawn from a low-income female population: women using a family planning clinic for primary care. Exercise, social support, mental health and other personal characteristics were measured and held constant in order to determine the independent effect of different sources of personal stress on obesity.

## Methods

A cross-sectional survey of primary care patients (adult non-pregnant women) attending one of five Planned Parenthood clinics in the Panhandle of Texas was conducted. The Amarillo Institutional Review Board granted exempt status to the study because no protected health information was collected. Planned Parenthood provide a valuable and unique sampling frame because it supplies basic primary care to low-income women in this area, including birth control, but not abortions.

Eligibility for the study was limited to patients who were over age 18 and not pregnant. Questionnaires were placed on a table in waiting areas, with a poster inviting participation. Clinic staff also handed out survey forms. Subjects placed the completed forms in a sealed box. Sealed boxes containing survey forms were returned to Texas Tech for data entry. Microsoft Access was used for data entry.

Five hundred forms were distributed. Twenty surveys were returned by persons ineligible for study participation and were excluded from the sample. The final data set was comprised of 345 subjects. Computing the response rate as completed returns divided by eligibles (345/(500-20) produces a participation rate of .719. Complete cases were available from 274 subjects for the multivariate analysis.

### Measures

The dependent variable was obesity. Body mass index (BMI) was computed as weight in pounds times 703 divided by height in inches squared. BMI greater than 30 was classified as obese, and made up about 20 percent of the sample. Cases with missing heights and weights were dropped.

Independent variables were stress, social support, age, race/ethnicity, education, income, number of persons in the home, exercise, marital status, anxiety, and depression. Key instruments are discussed below.

Health items were taken from the Duke Health Profile [9]. Mental health was measured in terms of feeling depressed or sad and nervousness. Possible responses for all of the mental health items were "Yes, describes me exactly", "Somewhat describes me," or "No, doesn't describe me at all."

Stress and social support items were taken from the Duke Social Support and Stress Scale (DUSOCS). The DUSOCS contains items addressing personal support from various sources. A person who stresses the respondent is defined as one who causes problems or makes life more difficult [10]. Respondents were asked how much they were stressed by spouses, parents and children. Possible responses were "None," "Some," "A Lot," and "There is No Such Person". Categories were combined into 'none' versus 'a lot or some.'

Exercise was measured in terms of times per week (none, one day, two days, three days, four days, more than four). Age, number of persons in the home, race/ethnicity (non-Hispanic white, Hispanic, other), marital status, and educational level (less than high school, high school or GED, more than high school) were used to control for demographic differences among subjects. The median age was 25. Age was categorized as 18–21, 21–30 or 31 or over. Breaks in the age distribution were made at the first and third quartile.

### Statistical analysis

Chi-square tests were used to test for the relationship between each independent variable and obesity. Variables that were significant at p < .10 in univariate tests were included in a multiple logistic regression analysis. EpiInfo 3.2.2 was used for data analysis.

## Results

Nearly half of respondents were classified as being obese (47.8 percent). About half of the respondents reported 'a lot or some' stress from parents or a spouse. 'A lot or some' stress from a child was experienced by about 40 percent. More than three-fourths of respondents said they receive 'a lot or some' support from a spouse or parent. About sixty percent received 'a lot or some' support from a child. Over 90 percent reported 'a lot or some' support from a friend.

Table 1 shows the results of the univariate chi-square tests. Of the three stress variables, only parent stress met the selection criterion for inclusion in the logistic regression model (p = .0988). Support from parents was marginally related to obesity (p = .0542) while support from a child was significantly to obesity (p = .0390).

**Table 1:** Psychosocial Risk Factors and Percent Obese in Family Planning Clinics (Chi-square tests)

	Pct Obese	Pct Not Obese	p
Overall	47.8	52.2	
Nervous			.6064
None	46.7	53.3	
Some	45.2	54.8	
A lot	57.1	42.9	
Depression			.1944
None or some	45.6	54.4	
A lot	59.4	40.6	
Stress from Parents			.0988
None	52.8	47.2	
A lot or some	42.7	57.3	
Stress from Spouse			.8084
None	49.1	50.9	
A lot or some	46.8	53.2	
Stress from Child			.1285
None	44.6	55.4	
A lot or some	54.2	45.8	
Support from Spouse			.1607
None	55.4	44.6	
A lot or some	45.6	54.4	
Support from Child			.0390
None	39.5	60.5	
A lot or some	42.2	47.8	
Support from Parents			.0542
None	61.7	38.3	
A lot or some	45.2	54.8	
Support from Friend			3472
A lot or some	45.9	54.1	
None	57.1	42.9	

The sample was comprised of relatively young women, with most being under 30 years of age. Most respondents lived with two or more other people and most had high school degrees. More than one-fourth of respondents were married. Over one-fourth were Hispanic and over half were non-Hispanic White. Most had incomes under $30,000 per year. Over 35 percent got no exercise at all.

Table 2 shows the results of univariate chi-square tests for the demographic variables and for exercise. Obesity differed significantly by the number of persons in the home (p = .0047), level of education (.0060), income level (p = .0328), and marital status (p = .0183). Over 58 percent of married respondents were obese, compared to 42.5 percent of unmarried persons. Person who lived alone were much less likely to be obese than persons who lived with four or more people (32.5 percent vs 64.8 percent). Over sixty percent of those lacking high school education were obese, whereas only about 40 percent of those who had more than a high school education were obese. The $10–20,000 income category had the lowest percent obese (36.1).

**Table 2:** Other Risk Factors and Percent Obese in Family Planning Clinics (Chi-square tests)

	Pct Obese	Pct Not Obese	p
Days of Exercise per Week			.3857
None	49.6	50.4	
One	57.1	42.9	
Two	53.4	46.6	
Three	39.6	60.4	
Four	31.8	68.2	
Five or more	41.7	58.3	
Missing	54.5	45.5	
Number of persons in home			.0047
None	32.5	67.5	
One to three	44.7	55.3	
Four or more	64.8	35.2	
Education			.0060
Less than high school	63.6	36.4	
High school degree or equivalent	55.3	44.7	
More than high school	39.7	60.3	
Race/Ethnicity			.3747
White, non-hispanic	44.6	55.4	
Hispanic	51.1	48.9	
Other	54.8	45.2	
Income			.0328
Less than $10,000	54.2	45.8	
$10–20,000	36.1	63.9	
$20–30,000	56.4	43.6	
Over $30,000	46.3	53.7	
Marital Status			.0183
Married	58.3	41.7	
Other	42.5	57.5	
Age			.3857
Less than 21	49.2	50.4	
21–30	44.6	55.4	
over 30	53.7	46.3	

'Some' or 'a lot; of nervousness was reported by about one-third of respondents, while over one-third said they were depressed 'some' or 'a lot'. Neither depression nor anxiety was retained for use in the multivariate model, since significance levels were below .10.

Variables that were significant at p < .10 were included in the multiple logistic regression model (see Table 3). Women who reported no support from parents had greater odds of being obese (adjusted odds ratio (AOR) = 2.17, p = .0420). Stress from parents and support from a child had no independent relationship with obesity. Persons who lived in homes of four or more were more likely to be obese (AOR = 4.05, p = .0089). Being in the $10,000 to $20,000 income category lowered the odds of obesity in comparison to the under $10,000 category (AOR=.4864, p = .0267).

**Table 3:** Unconditional Logistic Regression of Obesity in Family Planning Clinics (N = 274)

Variable	Odds Ratio (Conf. Interval)	P
Stress from parents		
(none vs a lot or some)	1.2427 (.73–2.13)	.4294
Support from child		
(none vs a lot or some)	.8949 (.51–1.57)	.6994
Support from parent		
(none vs a lot or some)	2.1710 (1.03–4.58)	.0420
Number in home		
One to three vs none	1.8413 (.80–4.24)	.1518
Four or more vs none	4.0503 (1.42–11.55)	.0089
Income		
$10–20 vs less than 10	.4864 (.26–.92)	.0267
$20–30 vs less than 10	1.1426 (.54–2.40)	.7248
over $30 vs less than 10	.4945 (.20–1.19)	.1176
Marital status		
Other vs married	.6095 (.32–1.16)	.1340
Education		
High school vs less	1.1435 (.43–3.07)	.7900
More than HS vs less	.7489 (.29–1.91)	.5439

Comparisons of cases with missing obesity information to cases with complete obesity information revealed no significant differences in regard to age, marital status, or income. However, ethnicity and education were significantly different for persons who were missing obesity information. Missings were more likely to be Hispanic or other than non-Hispanic white. Missings also were less likely to have a high school degree or higher.

## Discussion

According to our univariate analysis, the profile of an obese woman in this low-income population is having a large family, less than a high school education, and being married. They also were more likely to fall into income groups above or below the $10,000 to $20,000 range. Variables assessing stress from various sources were not significant at p < .05. Multivariate analysis revealed that receiving no support from parents was independently related to higher rates of obesity, while women in the $10,000 to $20,000 income category were less likely to be obese. The reasons for the income differences are not clear, though varying access to food assistance may offer a partial explanation. Additional investigation of the relationship between diet and income among low-income women is needed.

The findings of this study should be treated with caution since it is based on a convenience sample and may not be representative of the population from which it was drawn. In addition, the response rate was not optimal and also a number of cases were dropped from the analysis due to missing information, which reduced statistical power and could have biased our conclusions. However, since the sample was not randomly selected we cannot be sure that dropping cases with missing data made the sample less representative of the low-income female primary care population. An additional limitation of the study was its cross-sectional in design which does not allow for proving causal relationships. Because of these limitations, our results must be considered suggestive rather than definitive. Nevertheless, the findings may be important to primary care physicians, epidemiologists and others who study the determinants of obesity in clinic populations.

We could not demonstrate a significant relationship between self-reported stress and obesity in this relatively young, female population after adjustment for other variables. However, personal stress may have indirect effects on obesity, an issue not investigated in this study. Furthermore, the relationship between self-reported (perceived) stress with objective (psychological and physiological) measures of stress in this population group are unknown. Consistent with the findings of our study, Sammel et al did not find stress to be related to obesity in their study of women aged 35–47 [1].

Exercise was not significantly related to obesity in our data. Interestingly, Sammel et al also found no relationship between exercise and obesity 1]. Kaplan et al reported that physical activity was related to obesity in older Canadians, but their sample was quite large (N = 5,980) thus giving them more statistical power [3].

Obesity rates increased with age in our univarate analyses. This is consistent with what has been reported by other investigators [1,3]. We found no independent relationship with education or race, which agrees with Sammel but conflicts with other research [3,4,8]. This contradiction might be due to the fact that our sample was constrained to include primarily low-income women; obesity may be more strongly related to income than race, ethnicity or educational level.

We could not show self-assessed depression to be predictive of obesity. Several other investigators have examined this issue, with some seeing obesity as a consequence of depression and others regarding depression to be a result of obesity. In a large study by Carpenter et al [11], increased BMI in women was associated with major depressive disorder as diagnosed in a structured interview using DSM-IV criteria. A study of the National Health and Nutrition Examination Survey (NHANES) also found that obesity was related to depression. Furthermore, Sammel et al reported that weight gain was related to baseline depression, providing some support that depression may be a cause rather than a consequence of obesity [2]. Goodman and Whitaker studied the development and persistence of adolescent obesity and found that depressed mood at baseline was an independent risk factor of persistent obesity [4]. Obesity may also increase the risk of depression in women due to stigma and social isolation related to obesity, particularly among women in western cultures. Since our depression variable was drawn from a single question, it may have contained too much measurement error to permit it to achieve statistical significance in our data set.

The reasons why large families increase the risk of obesity are not entirely clear. One obvious mechanism is that, since women traditionally prepare meals, they may have more frequent opportunities to consume food and households with large families are more likely to have greater volumes of food available.

## Conclusion

The research question for this paper was about the risk factors for obesity in a low-income female population in a single community. Our study differs from some other studies of obesity by its inclusion of several types of personal stress as well as social support and mental health measures. We were able to show that personal stress, as defined and measured in this study, was not an important risk factor for obesity in this population group.

A limitation of this study is that it does not address occupational stress. Work-related stress has been shown to be related to the health of employees [12-14]. However, since many of the subjects in this sample were not employed, it was not feasible to test hypotheses regarding the relationship between work stress and obesity. Another limitation of the study is its cross-sectional design, which precludes making firm conclusions regarding causality.

Despite these limitations, our findings have significance for public health practice related to weight control. Health promotion programs that seek to educate and encourage healthier eating behaviors in low-income female populations should focus on women who are not receiving support from their parents and have large families of their own. In addition, income and eligibility for food assistance may affect dietary practices in unexpected ways, so primary care providers should explore this issue.

## Competing interests

None declared.

## Authors' contributions

JR planned the study, organized the survey, and wrote the first draft of the results. BR wrote the section addressing mental health and obesity.

## Pre-publication history

The pre-publication history for this paper can be accessed here:


